# Electronic medical record-based model to predict the risk of 90-day readmission for patients with heart failure

**DOI:** 10.1186/s12911-019-0915-8

**Published:** 2019-10-15

**Authors:** Bo-yu Tan, Jun-yuan Gu, Hong-yan Wei, Li Chen, Su-lan Yan, Nan Deng

**Affiliations:** 1 Division of Clinical Pharmacy, The First Affiliated Hospital of Hunan Normal University (Hunan Provincial People’s Hospital), Changsha, Hunan 410005 People’s Republic of China; 2 Division of Pharmacy, College of Medicine, Hunan Normal University, Changsha, Hunan 410013 People’s Republic of China; 3 Cardiovascular Department, The First Affiliated Hospital of Hunan Normal University, Changsha, Hunan 410005 People’s Republic of China

**Keywords:** Heart failure, 90-day readmission, Risk, Predicate

## Abstract

**Background:**

Several heart failure (HF) risk models exist, however, most of them perform poorly when applied to real-world situations. This study aimed to develop a convenient and efficient risk model to identify patients with high readmission risk within 90 days of HF.

**Methods:**

A multivariate logistic regression model was used to predict the risk of 90-day readmission. Data were extracted from electronic medical records from January 1, 2017 to December 31, 2017 and follow-up records of patients with HF within 3 months after discharge. Model performance was evaluated using a receiver operating characteristic curve. All statistical analysis was done using R version 3.5.0.

**Results:**

A total of 350 patients met the inclusion criterion of being readmitted within in 90 days. All data sets were randomly divided into derivation and validation cohorts at a 7/3 ratio. The baseline data were fairly consistent among the derivation and validation cohorts. The variables most clearly related to readmission were logarithm of serum N-terminal pro b-type natriuretic peptide (NT-proBNP) level, red cell volume distribution width (RDW-CV), and Charlson comorbidity index (CCI). The model had good discriminatory ability (C-statistic = 0.73).

**Conclusions:**

We developed and validated a multivariate logistic regression model to predict the 90-day readmission risk for Chinese patients with HF. The predictors included in the model are derived from electronic medical record (EMR) admission data, making it easier for physicians and pharmacists to identify high-risk patients and tailor more intensive precautionary strategies.

## Background

As the final stage of cardiovascular disease, heart failure (HF) is the leading cause of hospitalization for people aged over 65 years [1]. There are 4.5 million patients with HF in China, an incidence rate of about 0.9% [2]. With an associated high risk of mortality and readmission, the immediate period after hospital discharge after HF has been defined as a “vulnerable period” that generally lasts 2–3 months [1, 3, 4]. The prognosis of advanced HF is worse than that of some solid tumors and myocardial infarction. In severe cases, the 5-year survival rate is less than 20% [5]. In 2012, $5.42 billion was spent on HF treatment in China, which was the highest of any low or middle-income country. That expense accounted for 5.01% of global costs for HF medical care, increasing the associated social economic burden [6]. Facing this situation, it is necessary to develop a predictive model to identify patients with HF at high risk of readmission.

Existing models were mainly devised in developed countries to predict the 30-day risk of readmission after HF, and most of them have performed moderately [7–9]. The model developed by Huynh QL et al. has the best predictive power (C-statistic = 0.85) for 30-day HF readmission and mortality [10]. However, the discriminatory power of these models in the Chinese population with HF remains to be verified. Owing to ethnic and medical environmental differences and low per capita income, many regions lack basic medical facilities. For example, chest pain centers in China developed 20 years later than those in the United States [11]. Chinese patients with HF do not tend to be readmitted in the short term after their last discharge until their condition becomes unbearable. Compared with a 30-day period, the 90-day period after discharge may be more valuable for observation of readmission during the vulnerable phase among real-world Chinese patients with HF [12]. However, most studies on 90-day readmission have only identified risk factors associated with lack of recovery of activities of daily living and changes in medication regimen complexity [13, 14]. Only the model by Huynh QL has predicted 90-day HF readmission, and it has shown poor discriminatory power (C-statistic = 0.65) [10]. Moreover, some important factors in HF diagnosis, such as N-terminal pro b-type natriuretic peptide (NT-proBNP) and red cell volume distribution width (RDW-CV), were not analyzed in many previous studies. Further, there is currently no effective prediction model that can effectively manage Chinese patients with HP who have a high risk of readmission.

The study aimed to develop a risk model with convenient usage to identify HF patients at high risk for HF readmission within 90 days of initial discharge.

## Methods

### Data source and extraction

**Fig. 1:**
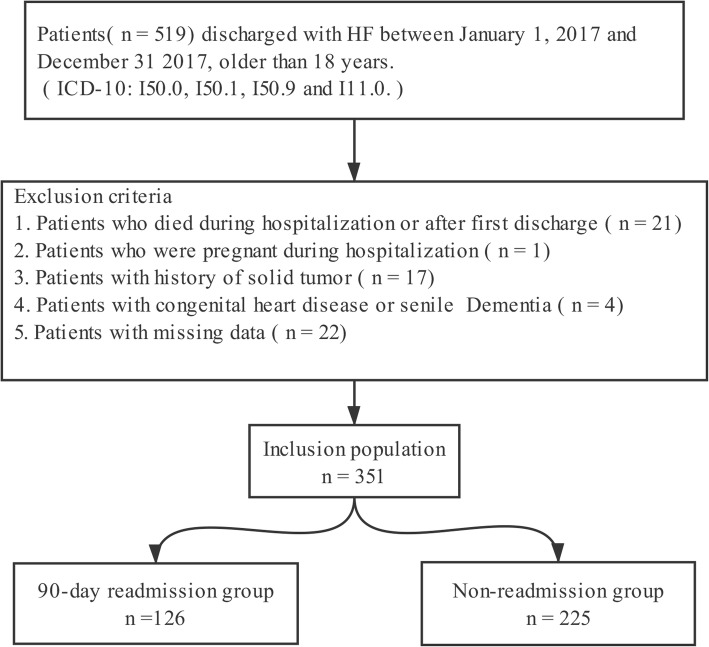
Patient selection flow chart

Clinical data for this study were obtained from the Hunan Provincial People’s Hospital. All patients hospitalized for HF with discharge dates from January 1, 2017 to December 31, 2017 were included. Available information included demographics, admission diagnosis, past medical history, comorbidities at admission, medical process, medications prescribed at discharge, complete blood count, and laboratory tests. Other extracted data included number of admissions, length of stay, medical insurance, and New York Heart Association (NYHA) class at admission. According to the 10th revision of the International Statistical Classification of Diseases, patients with a main diagnosis of HF at admission were identified based on the codes I50.0, I50.1, I50.9, and I11.0. Adults were defined as patients aged 18 years or older. Patients with solid tumors, death in hospital or within 3 months after first discharge, pregnancy, congenital heart disease, senile dementia, or missing data were excluded. Figure 1 shows this study’s patient selection process. This study was approved by the Ethics Committee of the First Affiliated Hospital of Hunan Normal University (Hunan Provincial People’s Hospital) (approval no. 2018–35). Informed consent was exempted by the Ethics Committee because of the retrospective nature of this research. Patient records/information were anonymized and de-identified prior to analysis.

### Study design

A total of 52 variables were included in the study as potential prognostic factors. Patients who had records of unplanned admissions no less than twice within 90 days with principle diagnoses of HF were defined as the readmission group. The non-readmission group was composed of the patients who did not require readmission with HF within the same period. Patients with HF have a high and increasing prevalence of comorbidities [15]. The prognostic value of comorbidities was observed using the Charlson Comorbidity Index (CCI) [16]. To maintain independence of observations, if patients underwent laboratory tests and blood tests more than once during the study period, only the results from the first laboratory report were analyzed.

### Outcome

The primary outcome of this study was readmission within 90 days after first admission for HF. The data from electronic medical records and the results of our follow-up were used to measure this outcome.

### Follow-up

At the first and third months after first discharge, follow-up visits were performed by cardiovascular doctors and clinical pharmacists involved in this study. Medication usage and patient event registration were recorded during these visits, and the investigators reconfirmed whether the patient was hospitalized in other community medical institutions or hospitals after first discharge. This information was used to divide the two groups correctly.

### Statistical analysis

Continuous variables were presented as mean and standard deviation (SD) for normally distributed variables and compared using Student’s t-tests followed by the Levene test to ensure equality of variances. For non-normally distributed data, median and interquartile range (IQR) were presented, and the Wilcoxon rank-sum test was used. Categorical data were expressed as numbers and percentages and compared by Chi-square tests. Related model parameters were presented as adjusted odds ratios (OR), corresponding two-sided 95% confidence intervals (CIs), and *p*-values for the predictors. The Hosmer-Lemeshow test was used to observe the fitness of the model. All statistical analysis was performed using R version 3.5.0 (R Foundation for Statistical Computing; Vienna, Austria; ISBN 3–000051–07-0, http://www.R-project.org). *p* values < 0.05 were considered as statistically significant.

### Modeling and validation

The full dataset was split 7:3 into derivation and validation cohorts by random sampling. Logistic regression was used to create the derivation cohort, and the predictors found in univariate analysis of the derivation cohort were used to determine the predictors for readmission. Variable reassignment and conversion were performed to improve the model by comparing each model. The variance inflation factor (VIF) was used to test for multicollinearity among the predictors in models to ensure the independence of each variable. Values of VIF over 4.0 were considered to indicate an interaction among predictors [17].

The final risk prediction model was evaluated in terms of discriminatory power among the validation cohort. Discriminatory power was used as a measure of the model’s ability to distinguish between readmitted and non-readmitted patients. In addition, the discriminative power of the prediction model was evaluated by C-statistics based on receiver operating characteristic (ROC) curves. C > 0.7 indicates that the model has reasonable discriminatory power.

## Results

### Characteristics of study cases

A total of 519 patients were screened, and 350 met the inclusion criterion of admission for chronic HF. The demographic and clinicopathologic distributions of both the derivation and validation cohorts are shown in Table 1. There were no significant differences between the derivation and validation cohorts. Overall cardiac-related readmission occurred in 127 (36.3%) patients across the full dataset.

**Table 1:** Characteristics of included HF patients between derivation and validation cohort

Characteristic	Derivation cohort (*n* = 246)	Validation Cohort (*n* = 104)	*p-*value
Age (years) [mean ± SD]	67.7 ± 12.3	69.0 ± 12.9	0.36
Men [n (%)]	153 (62.2)	63 (60.6)	0.87
NYHA III/IV at Discharge [n (%)]	203 (82.5)	89 (85.6)	0.78
Infection [n (%)]	61 (24.7)	24 (23.1)	0.84
Ischemia [n (%)]	66 (26.8)	36 (34.6)	0.18
Cardiac overload [n (%)]	43 (17.5)	11 (10.6)	0.14
Hypertension [n (%)]	106 (43.1)	40 (48.1)	0.45
Arrhythemia [n (%)]	94 (38.2)	44 (42.3)	0.55
Myocardial infarction [n (%)]	51 (20.7)	19 (18.2)	0.70
Cerebral infarction [n (%)]	38 (15.4)	21 (20.2)	0.35
Chronic kidney disease [n (%)]	81 (32.9)	36 (34.6)	0.86
Chronic liver disease [n (%)]	49 (19.9)	27 (26.0)	0.27
Type 2 diabetes [n (%)]	69 (28.5)	36 (34.6)	0.31
COPD [n (%)]	22 (9.0)	12 (11.5)	0.58
Previous PCI [n (%)]	34 (13.8)	19 (18.3)	0.37
Left Ventricular Ejection Fraction [Median (IQR)]	36.5 (27.0, 54.0)	35 (26.0, 49.3)	0.24
Charlson Comorbidity Index [Median (IQR)]	2 [1, 2]	2 [1, 3]	0.80
Length of hospital stay (days) [Median (IQR)]	9 (7, 11.75)	9 (6.0, 11.0)	0.26

*COPD* chronic obstructive pulmonary disease, *PCI* percutaneous coronary intervention, *IQR* interquartile range

Univariate analysis of the derivation cohort identified nine factors as significantly affecting readmission rates, including NYHA class, NT-proBNP, RDW-CV, urea nitrogen, cardiac troponin I (cTnI), CCI, and hemoglobin (Hgb). The characteristics of readmission are shown in Table 2. Table 3 shows the medications prescribed at patients’ discharge. Different from the non-readmission group, less angiotensin-converting enzyme inhibitor (ACEI) and angiotensin receptor blockers (ARBs) but more nitrates were used in the readmission group (*p* < 0.05). Table 4 summarizes medications prescribed within 90 days after first discharge; these results did not significant differ between groups.

**Table 2:** Characteristics of HF patients in derivation cohort stratified by readmission status

Characteristic	Readmission (*n* = 86)	Non-readmission (*n* = 160)	*p-*value
Age (years) [Mean ± SD]	69.9 ± 10.4	66.4 ± 13.1	0.019^*^
Men [n (%)]	59 (68.6)	84 (61.3)	0.31
NYHA rank at discharge (III/IV) [n (%)]	77 (89.5)	139 (86.9)	0.034^*^
NT-proBNP (pg/mL) [Median (IQR)]	5963 [3031, 11,269]	4874 [1607, 5732]	< 0.001^*^
Ft3 (pmol/L) [Median (IQR)]	3.6 [3.2, 4.2]	3.7 [3.2, 4.3]	0.42
Ft4 (pmol/L) [Median (IQR)]	16.9 [15.07, 18.8]	17.5 [15.1, 19.8]	0.27
Tsh (uIU/mL) [Median (IQR)]	3.0 [1.3, 3.9]	2.4 [1.2, 4.1]	0.91
White Blood Cell Count (×10^9^/L) [Median (IQR)]	6.5 [5.0, 7.7]	6.4 [5.2, 8.0]	0.61
Hemoglobin (g/L) [Mean ± SD]	112.4 ± 21.3	121.6 ± 20.6	0.001^*^
RDW-CV (%) [Median (IQR)]	14.1 [13.3, 15.6]	13.7 [12.9, 14.7]	0.01^*^
RDW-SD (fL) [Median (IQR)]	47.7 [44.7, 51.4]	46.7 [43.3, 49.5]	0.098
Platelet (×10^9^/L) [Median (IQR)]	170 [142, 221]	166 [130, 209]	0.31
Meam platelet volume (fL) [Mean ± SD]	10.7 ± 1.3	10.9 ± 1.3	0.17
Glucose (mmol/L) [Median (IQR)]	5.0 [4.2, 6.5]	4.6 [4.2, 5.6]	0.10
Total Cholesterol (mmol/L) [Median (IQR)]	3.5 [2.9, 4.0]	3.4 [2.8, 4.2]	0.47
Triglyceride (mmol/L) [Median (IQR)]	1.3 [1.0, 1.4]	1.1 [0.8, 1.5]	0.47
High-density lipoprotein (mmol/L) [Median (IQR)]	0.93 [0.8, 1.1]	0.97 [0.8, 1.1]	0.30
Low-density lipoprotein (mmol/L) [Median (IQR)]	2.0 [1.7, 2.5]	1.9 [1.5, 2.6]	0.81
Blood urea nitrogen (mmol/L) [Median (IQR)]	8.8 [5.9, 10.8]	6.2 [4.4, 8.3]	< 0.001^*^
Cardiac troponin I (ng/mL) [Median (IQR)]	0.043 [0.022, 0.069]	0.026 [0.013, 0.047]	< 0.001^*^
Alanine aminotransferase (u/L) [Median (IQR)]	19.0 [12.9, 32.2]	21.5 [12.6, 32.5]	0.27
Aspartate transaminase (u/L) [Median (IQR)]	26.2 [19.1, 37.9]	25.9 [19.5, 39.2]	0.84
Infection [n (%)]	20 (23.2)	41 (26.3)	0.79
Ischemia [n (%)]	21 (24.4)	45 (28.1)	0.63
Cardiac overload [n (%)]	17 (11.6)	26 (16.2)	0.61
Hypertension [n (%)]	35 (40.7)	71 (44.3)	0.67
Arrhythmia [n (%)]	32 (37.3)	62 (38.8)	0.86
Myocardial infarction [n (%)]	17 (19.8)	34 (21.3)	0.91
Cerebral infarction [n (%)]	10 (11.6)	28 (17.5)	0.30
Chronic kidney disease [n (%)]	37 (43.0)	44 (27.5)	0.019^*^
Chronic liver disease [n (%)]	16 (18.6)	33 (20.6)	0.83
Type 2 diabetes [n (%)]	22 (25.6)	47 (29.3)	0.63
COPD [n (%)]	12 (14.0)	10 (6.3)	0.07
Previous PCI [n (%)]	9 (10.5)	25 (15.6)	0.35
Left Ventricular Ejection Fraction [Median (IQR)]	35.0 (25.0, 53.0)	37.0 (28.0, 56.0)	0.24
Charlson Comorbidity Index [Median (IQR)]	2 [1.0, 3.0]	2 [1.0, 2.25]	< 0.01^*^
Length of hospital stay (days) [Median (IQR)]	9 (7.0, 12.75)	9 (7.0, 11)	0.83
Medical insurance [n (%)]	61 (73.6)	109 (65.4)	0.65

*NYHA* New York heart association functional class, *NT-proBNP* N-terminal pro b-type natriuretic peptide, *FT3* free triiodothyronine, *FT4* free thyroxin, *TSH* thyroid-stimulating hormone, *RDW-CV* red blood cell distribution width coefficient of variation, *COPD* chronic obstructive pulmonary disease, *PCI* percutaneous coronary intervention, *IQR* interquartile range

^*^Denotes *p* < 0.05, Differences between readmission group and non-readmission group were tested for statistical significance

**Table 3:** Medications during hospitalization

Characteristic	Readmission (*n* = 86)	Non-readmission (*n* = 160)	*p-*value
ACEs/ARBs [n (%)]	61 (73.6)	135 (85.8)	0.024^*^
Beta blockers [n (%)]	61 (71.9)	124 (78.7)	0.27
Ca channel blockers [n (%)]	43 (45.4)	60 (40.4)	0.47
Thiazide [n (%)]	12 (10.7)	11 (7.8)	0.75
Aldosterone blockers [n (%)]	57 (68.6)	95 (72.4)	0.60
Loop diuretics [n (%)]	41 (60.3)	61 (64.6)	0.48
Nitrates [n (%)]	66 (79.3)	107 (65.2)	0.023^*^

*ACEs/ARBs* angiotensin converting enzyme inhibitors or angiotensin II receptor blockers

^*^Denotes *p* < 0.05, Differences between readmission group and non-readmission group were tested for statistical significance using Pearson’s Chi-square test

**Table 4:** Medications use registered by following up

Characteristic	Readmission (*n* = 86)	No readmission (*n* = 160)	*P* value
ACEs/ARBs	62 (72.1%)	96 (60.0%)	0.081
Beta blockers	58 (67.4%)	122 (76.3%)	0.182
Ca channel blockers	19 (22.1%)	30 (18.8%)	0.647
Thiazide	2 (2.3%)	9 (5.6%)	0.384
Aldosteroneblockers	60 (69.8%)	107 (66.9%)	0.749
Loop diuretics	50 (58.1%)	98 (61.3%)	0.735
Nitrates	12 (14.0%)	30 (18.8%)	0.438

*ACEs/ARBs* angiotensin converting enzyme inhibitors or angiotensin II receptor blockers

### Model summary

Three variables eventually entered the model. Table 5 lists the effects of NT-proBNP, RDW-CV, and CCI on 90-day readmission, all of which were found to be significant. The final equation of the obtained prediction model is:

$$ Prob\ (readmission)=1/\left[1+\exp\ \left(9.589-1.521\times \log NT- proBNP-0.204\times RDW- CV-0.266 CCI\right)\right]. $$

**Table 5:** Logistic regression model for predict 90-day readmission risk of HF patients

Factor	Adjusted OR	95%CI	*p-*value
Log (NT-proBNP)	4.578	2.344–9.351	< 0.001
RDW-CV	1.226	1.022–1.480	0.029
CCI	1.305	1.063–1.611	0.011

*NT-proBNP* N-terminal pro b-type natriuretic peptide, *RDW-CV* red blood cell distribution width coefficient of variation, *CCI* Charlson comorbidity index

For convenience of clinical application of this model, risk score was defined as an independent variable replacing the risk factors. Thus, the above equation can be simplified to:

$$ Prob\ (readmission)=1/\left[1+\exp \left(-\mathrm{score}\right)\right]. $$

**Fig. 2:**
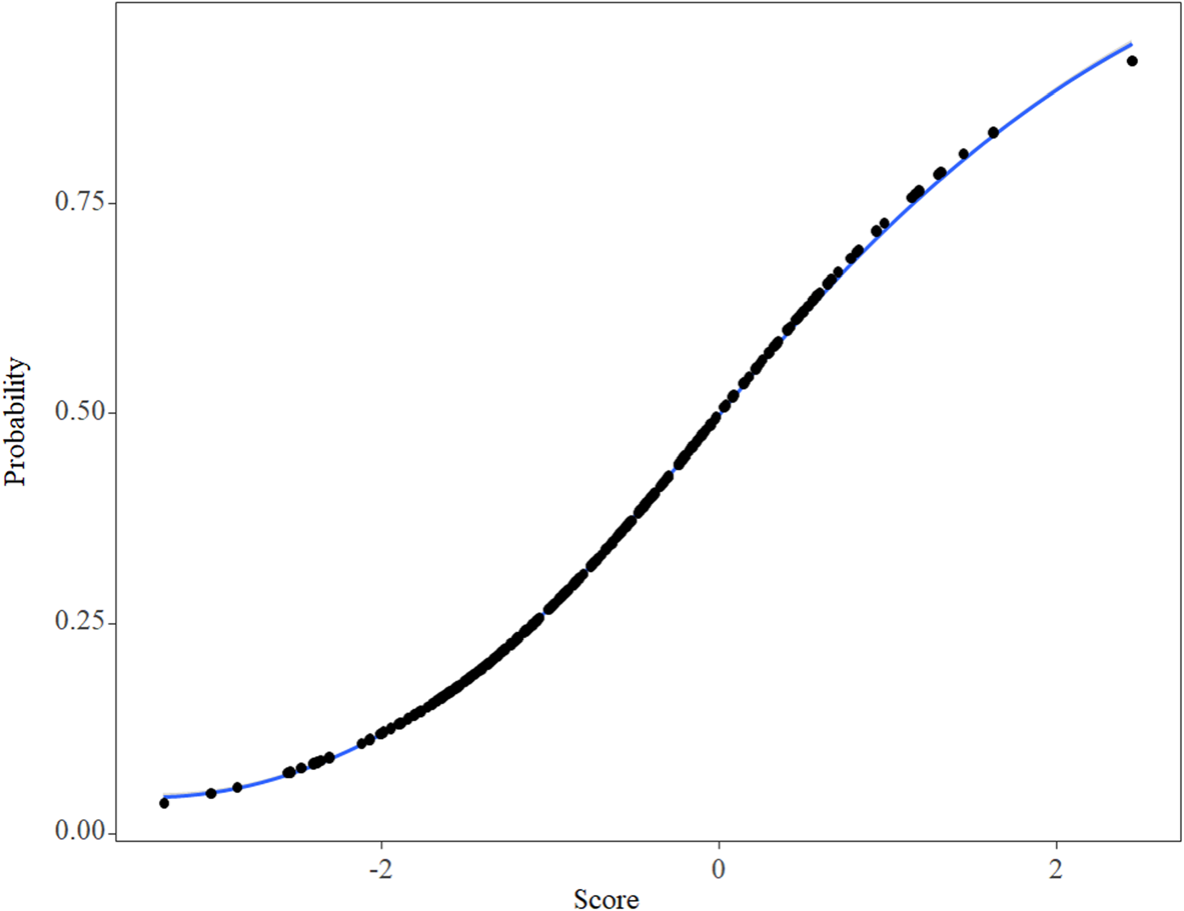
Risk prediction of 90-day readmission after initial admission for HF

Figure 2 shows a visualization of the correspondence between readmission risk and score. The VIF values of predictor variables in this model were all less than 1.1, indicating that there is no multicollinearity, and the Hosmer-Lemeshow test showed that the model has favorable fitness (*p* = 0.62).

### Discriminatory power of model

**Fig. 3:**
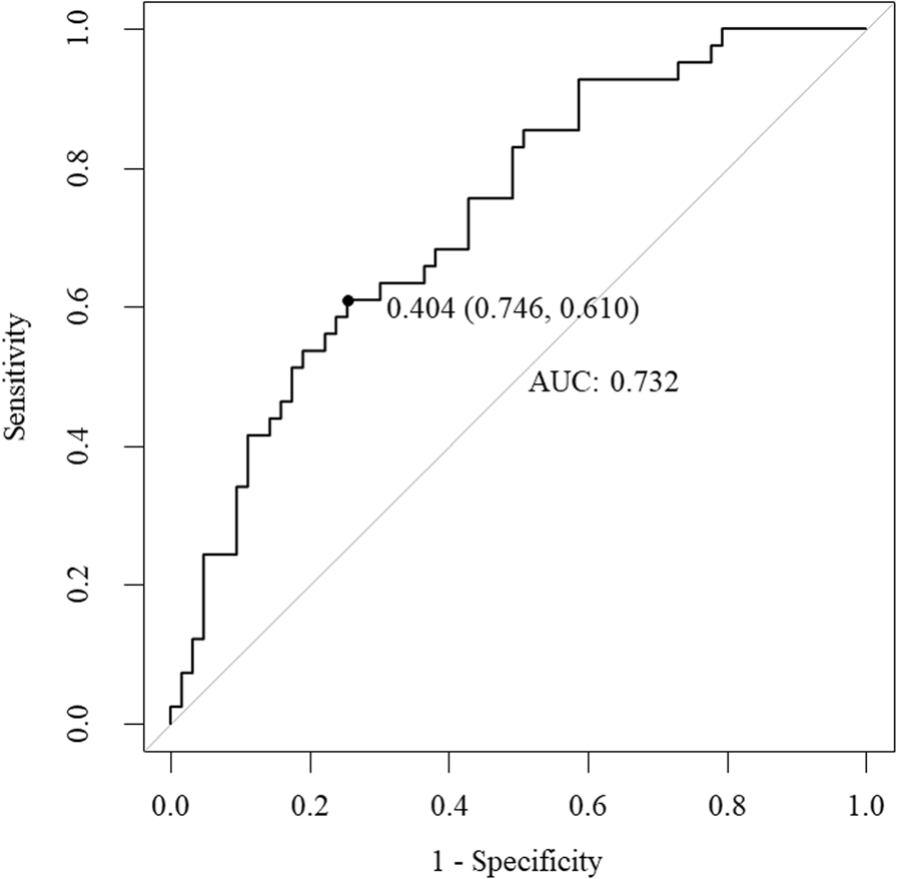
Discriminatory power of the model

**Fig. 4:**
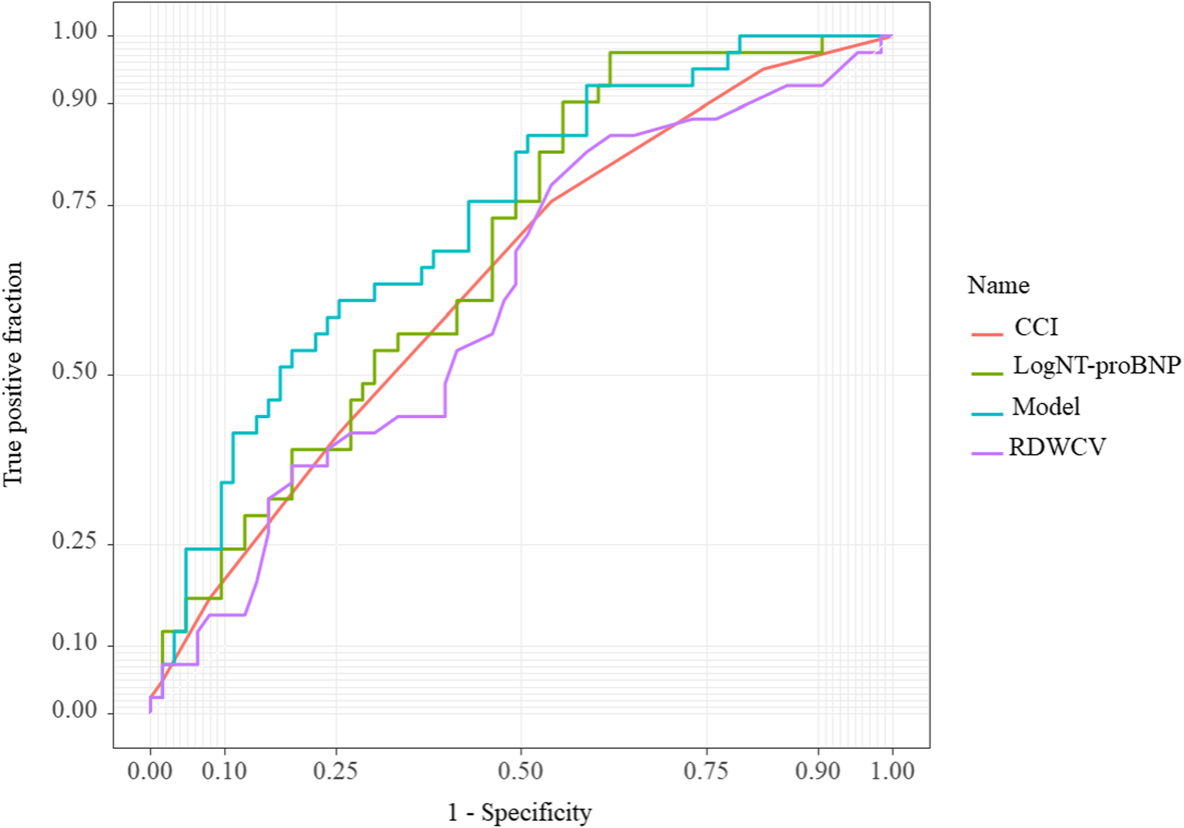
Comparison of receiver operating characteristic curves of four models

An ROC for the model is described in Fig. 3, showing that a predicted score of 40.4% risk (sensitivity 74%, specificity 61%) was optimal for 90-day readmission. Figure 4 depicts a model that shows better discriminatory power for 90-day readmission by comparing another single variable. The values of C-**statistic** were: Model (0.73) > NT-proBNP (0.68) > CCI (0.64) > RDW-CV (0.61).

## Discussion

Improving quality of life for patients with HF is still a challenge worldwide. Although there are already some HF risk models, they come from clinical studies with more stringent inclusion criteria or from countries with developed medical technologies. These models have certain limitations for the real-world population with HF in China. Herein, we report a risk model using EMR-based algorithms derived from data gathered from HF patients in China. Our models focused on readmission using clinical variables that are readily accessible to electronic health record systems.

During the modeling process, because NT-proBNP levels fluctuated too much, with the highest one reaching 35,000 pg/mL, logarithms were taken to compare whether a better model could be obtained. As some of the best diagnostic factors for HF, b-type natriuretic peptide (BNP) and NT-proBNP were closely related to left ventricular remodeling [17]. Limited reports have indicated that discharge BNP, with an area under the ROC curve close to 0.7, was the best biomarker to predict readmission of patients with HF within 60 days [18], but NT-proBNP was tested in our study before clinical interventions. Therefore, more research is needed to compare the prognostic value of NT-proBNP/BNP tested at two different time points of readmission with HF, even though it was the strongest predictor in our model. The prognostic value of RDW-CV in HF has also been confirmed in previous reports [19, 20]. Elevation of RDW is usually associated with increased destruction of red blood cells and Hgb, which was consistent with the Hgb levels in the readmission group. The RDW-CV values in both groups were within the normal medical reference range (RDW-CV: 11–16%). Simple observation of this variable from clinical blood test reports cannot be used to intuitively judge the risk of readmission, and the area under the curve of RDW-CV also confirmed its weak discriminatory ability (C-statistic = 0.61). Chinese patients with HF often lack self-management awareness, such that many comorbidities were prone to occurring. The median of CCI was 2 in both groups, suggesting that most patients with HF in China have no less than one comorbidity. In Table 2, only chronic kidney disease (CKD) showed a large incidence rate among the readmission group. However, the severity of comorbidities caused moderate CCI increase in the readmission group. Morbidity associated with moderate or severe CKD increase is seen as a key factor in the readmission group, and the same trends are manifested in chronic liver disease. Because China is the country with the largest tobacco production and consumption, and its air quality is among the worst in the world, its prevalences of chronic obstructive pulmonary disease (COPD) and CKD are elevated. A review of global COPD illustrates that the exact cause of COPD is unclear, but many studies have shown that the most important risk factor is tobacco smoke [21].

Currently, the outcome of other scoring models is primarily 30-day readmission, and some of these models combined with a range of clinical and non-clinical factors have achieved good discrimination. With a C-value of 0.73 on the validation set in this study, our model of 90-day readmission has good performance. Preliminary estimates show that its discriminatory power is similar to that of Huynh QL’s model for 30-day readmission with HF, which was validated on the data of 1046 patients [10]. However, excessive variables in their model may increase the complexity of their use case: especially for some patients with severe HF patients at the time of admission, it can be difficult to complete the three sets of psychological assessments employed by that model, making it impossible for the model to render rapid predictions. Kitamura M found that activities of daily living were related to readmission within 90 days in patients with HF using a Cox regression model [14], which obtained a good area under the curve of 0.78, but the adapted population of this model was exclusively aged over 65 years. Further, that model has not been externally validated, and therefore, its practicality is limited.

### Other variables of potential interest

Previous reports have indicated that age and NYHA class are independent risk factors for 30-day readmission among patients with HF [10, 22, 23], and both of these factors also showed between-group differences in our univariate analysis. However, these relations became weaker in multivariable analysis and did not add incremental value to the model’s discriminatory ability. Part of the reason may be that the age of patients with HF in China tends to be younger than that in the United States, Europe, and Japan, as confirmed by the largest multi-center, prospective HF registration study, China-HF [24]. China’s proportion of middle-aged patients may result in age having weaker prognostic value. In our study sample, the NYHA grades were mostly III–IV. This result may be related to the habits of Chinese patients, who are reluctant to go to the hospital without being critically ill. This particularity may be one of the reasons why the discriminatory ability of NYHA grade is not significant. Moreover, the statistical significance of BUN and CKD showed that the readmission group has worse renal function. Related studies have confirmed that the increase in BUN and poor renal function is associated with readmission in patients with HF [25, 26]. Poor renal function and anemia are related closely. On the one hand, reduced production of erythropoietin in the kidney is a cause of anemia [27]. On the other hand, the severity of anemia directly affects renal function [28]. According to the World Health Organization diagnostic criteria for anemia (Hgb level below 130 g/L in men and 120 g/L in women) [29], The lower Hgb level in readmission group is related to the above conclusion. cTnI, as a sensitive marker of myocardial injury, plays an important role in the diagnosis, progression, and prognosis of myocardial injury [30]. In terms of value for predicting heart failure, only Escribano D et al. analyzed the troponin T (TnT) level of the initial laboratory report at the time of admission [31], showing that TnT was independently associated with readmission with acute heart failure (AHF). The troponin investigated in our study is a different subunit TnI, which failed to increase model discrimination ability. Although the above variables, which indicated statistical significance in univariate analysis, were not included in the final model, Masahiro Kitamura also reported similar discoveries about age, NYHA class, and Hgb [14]. However, limited research has been conducted on these factors, and therefore, these potential variables require focus in future study.

The impact of medications has always been a complex issue. Other significant factors include usage of ACEI, ARBs, and nitrates during hospitalization. Previous reports confirmed that ACEI and ARBs can prevent hospitalizations for HF [32]. In our study, approximately 40% of patients had hypertension. Therefore, ACEI or ARBs were often used to treat ventricular remodeling and hypertension. Nitrates were usually used clinically as a vasodilator for angina pectoris or congestive heart failure. Only Richardson A has found that nitrates were associated with increased 30-day readmission rate for COPD and reduced ejection fraction in HF [33]. So far, relevant evidence about the effects of nitrates on post-discharge patients with HF is still lacking.

### Clinical implications and future perspectives

Application of this model may provide value by targeting interventions to patients with HF who are at the highest risk of readmission. With the increased demand for improvement of the quality of medical care in China [21], there is a need for more optimized web-based quality monitoring systems for HF, which will provide more evidence for clinicians to improve adherence.

### Limitations

This study has some limitations. First, this study was conducted at one facility with a small sample. To improve the model’s discriminatory power and apply it to the real world effectively, more data from multiple centers will be needed in the future. Moreover, our data did not include some important prognostic variables, such as hypersensitive C-reactive protein [34], because most of them were missing. Readmission is related not only to medication selection but also to the combination and dosage of medications. There are missing data on patient compliance, which may affect the robustness of the results. Thus, a web-based quality monitoring system for HF is needed in the future.

## Conclusion

We developed and validated a multivariate logistic regression model to predict the 90-day readmission risk for Chinese patients with HF. The predictors included in the model are derived from EMR admission data, making it easier for physicians and pharmacists to identify high-risk patients and tailor more intensive precautionary strategies. Further research is needed to validate the extension of the model and to improve its discriminatory power.

## Data Availability

The datasets used and/or analyzed during the current study are available from the corresponding author on reasonable request.

## Abbreviations

ACEI: Angiotensin-converting enzyme inhibitor
ARBs: Angiotensin receptor blockers
BNP: b-type natriuretic peptide
CCI: Charlson comorbidity index
CI: Confidence interval
CKD: Chronic kidney disease
COPD: chronic obstructive pulmonary disease
cTnI: Cardiac troponin I
CV: Coefficient of variation
HF: Heart failure
Hgb: Hemoglobin
IQR: Interquartile range
NT-proBNP: N-terminal pro b-type natriuretic peptide
NYHA: New York Heart Association
OR: Odds ratio
RDW: Red cell volume distribution width
ROC: Receiver operating characteristic curve
SD: Standard deviation
Tn: Troponin
VIF: Variance inflation factor
